# Free bone fragments are associated with the development of heterotopic ossification after hemiarthroplasty for femoral neck fracture: a retrospective observational study

**DOI:** 10.1007/s00590-024-04083-6

**Published:** 2024-08-30

**Authors:** Hiroyuki Yokoi, Yasuhiko Takegami, Satoshi Ochiai, Takumi Miyachi, Tetsuya Urasaki, Shiro Imagama

**Affiliations:** 1 Department of Orthopaedic Surgery, Nagoya University Graduate School of Medicine, 65 Tsurumai-Cho, Showa-Ku, Nagoya, 466-8550 Japan; 2 Department of Orthopaedic Surgery, Chutoen General Medical Center, 1-1 Shobugaike, Kakegaswa-Shi, Shizuoka-Ken, 436-8555 Japan

**Keywords:** Heterotopic ossification, Hemiarthroplasty, Observational study, Femoral neck fractures

## Abstract

**Purpose:**

Heterotopic ossification (HO) can occur after hemiarthroplasty (HA) for femoral neck fractures (FNF). This study aimed to investigate the frequency and factors contributing to the development of HO after HA.

**Methods:**

The study included data from 92 of 183 patients (26 male and 66 female) who sustained FNF and underwent HA between April 2019 and January 2022. HO was identified on postoperative radiographic images. Patient background, operative duration, blood loss, and presence of free bone fragments immediately after surgery were compared between the HO and non-HO groups. Statistical analyses included the independent-sample *t*-test for continuous variables and the chi-squared test for categorical variables. A multivariate logistic regression analysis was performed using HO as an objective variable.

**Results:**

HO occurred in 50 of the 92 (54%) patients. There were no statistically significant differences in patient backgrounds. Univariate analysis revealed significantly longer mean operative duration and greater blood loss in the HO group. Free bone fragments in the immediate postoperative period were observed in 29 of 50 (58%) patients in the HO group and in 3 of 42 (7.1%) patients in the non-HO group, a statistically significant difference. Logistic regression analysis revealed that the presence of free bone fragments was an independent explanatory factor for HO development.

**Conclusion:**

The presence of free bone fragments immediately after surgery may be significantly associated with the development of HO. Therefore, it is necessary to sufficiently remove such fragments during surgery because they may trigger HO.

## Introduction

Heterotopic ossification (HO) is defined as the formation of lamellar bone in non-osseous tissues, such as muscles and joint capsules. HO is a relatively common complication of hip surgery involving open reduction and internal fixation, total hip arthroplasty (THA), or hemiarthroplasty (HA) [1]. HO is typically asymptomatic; however, severe HO causes pain and limitation(s) of the hip range of motion, which in turn leads to decreased gait function [2, 3]. A recent study reported a 15–90% prevalence of HO after THA [4–6]. However, there are few data regarding HO in the femoral neck of patients who underwent HA. Ozturkmen et al. reported a 0% incidence of HO in 48 patients who were treated with cementless HA for femoral neck fractures (FNF) [7]. In contrast, Corrigan et al. investigated 80 patients aged > 60 years undergoing HA for femoral neck fractures and reported an HO incidence of 27% [8].

Several studies have reported that factors such as a history of HO, hypertrophic osteoarthritis, and ankylosing spondylitis are associated with the development of HO in patients with hip disease. In a systematic review comparing cemented and cementless HA, Elmenshawy et al. reported that HO was significantly associated with longer operative duration, greater blood loss, and the use of bone cement [9]. In addition, some studies have reported a relationship between bone debris (i.e., free bone fragment[s]) during surgery and HO, which introduces a signal from the site of injury in an environment conducive to the promotion of heterotopic bone growth [5, 10, 11]. However, the definition of free bone fragments is ambiguous, and there is no established assessment. Few studies have investigated the relationship between HO and free bone fragment(s), which can be identified using X-radiography. This single-center, retrospective study aimed to investigate the incidence of HO after HA in patients who sustained FNF and to identify factors influencing the development of HO, including the presence of free bone fragments.

## Materials and methods

### Study design and setting

This was a retrospective, observational study. All patients provided informed consent, and the study protocol was approved by the ethics review board of the authors’ hospital.

### Patient selection

**Fig. 1:**
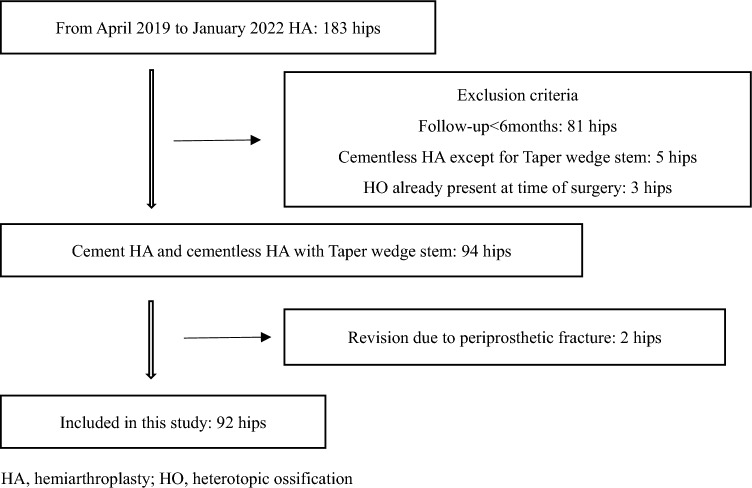
Study flowchart

The medical records of 183 patients who underwent HA for FNF between April 2019 and January 2022 were retrospectively reviewed. The inclusion criteria were cemented or cementless HA with at least 6-month follow-up period. Patients with HO at the time of surgery and those who underwent revision surgery were excluded. Finally, data from 92 patients were analyzed (Fig. 1).

### Data collection

Patient demographic data, including age, sex, body-mass index (BMI), pre-injury mobility level (graded as independent, cane ambulation, front-walker or frame ambulation, wheelchair, and bedridden), days to surgery, American Society of Anesthesiologists classification, operative duration, blood loss, and use of NSAIDs (loxoprofen sodium, 60 mg) during admission were obtained from electronic medical records housed in the authors’ hospital. The Parker Mobility Score (PMS) [12] before injury was also recorded, which was used to assess patient ambulatory status and walking ability, and was rated on a scale of 0 to 9.

### Diagnosis of HO

The frequency of HO and the stage of extension based on the Brooker classification [13] were evaluated using immediate postoperative radiographic images, and at 2, 12, and 24 weeks postoperatively. HO was defined as the detection of HO at any time point. The Brooker classification divides the extent of HO formation into four classes: class 1 (islands of bone within the soft tissues of the hip), class 2 (bone spurs originating from the pelvis or proximal end of the femur, leaving ≥ 1 cm between opposing bone surfaces), class 3 (bone spurs originating from the pelvis or proximal end of the femur, reducing the space between opposing bone surfaces to < 1 cm), and class 4 (apparent hip bone ankylosis). Brooker did not describe a “class 0” in the original study; however, subsequent studies using the Brooker classification have defined class 0 as the absence of radiographic HO [14].

### Radiographic evaluation

**Fig. 2:**
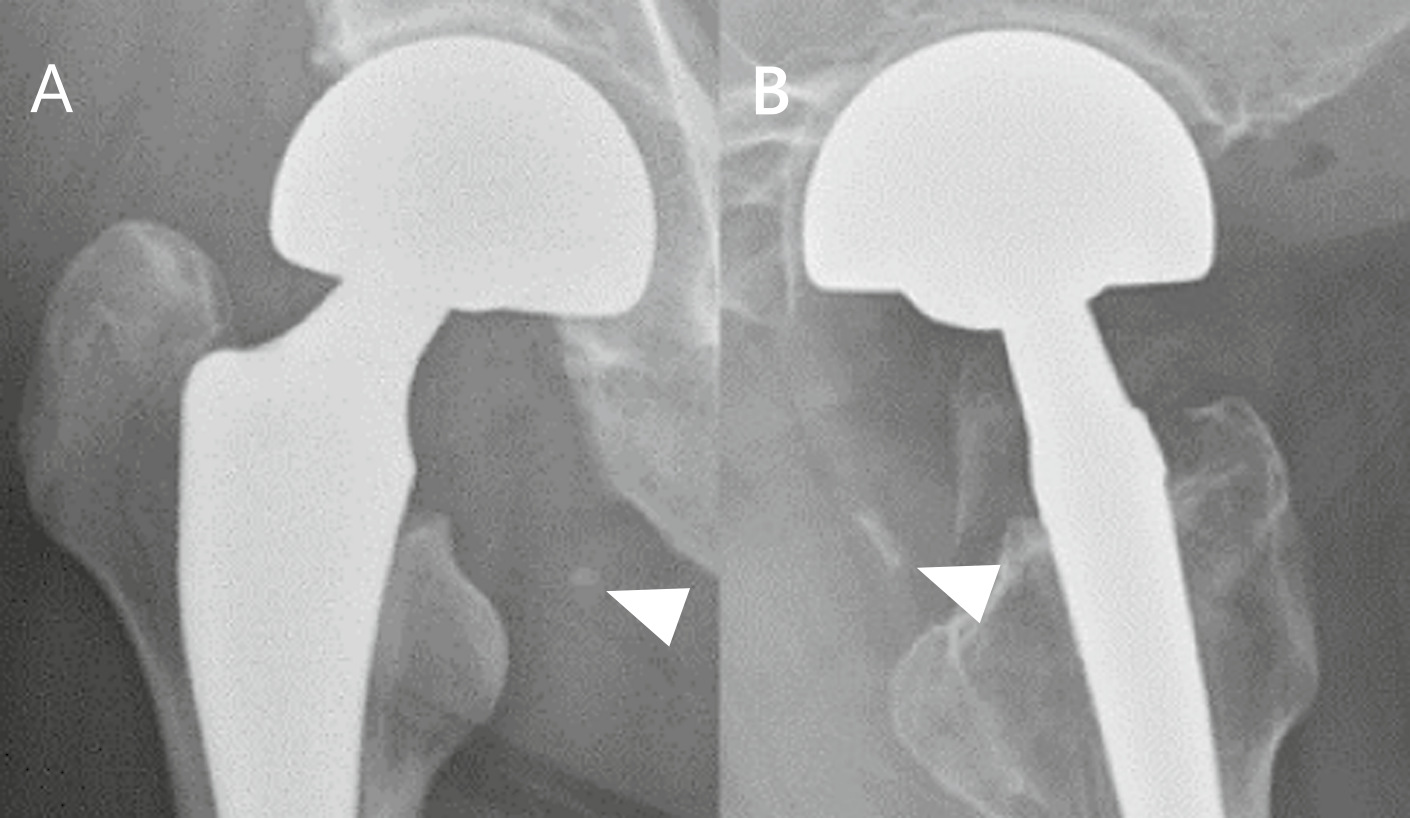
Free bone fragment immediately after surgery. **A** Anteroposterior radiograph. Arrowhead shows free bone fragment. **B** Lateral radiograph. Arrowhead shows free bone fragment

Anteroposterior and lateral views of radiographs captured postoperatively and at 2, 12, and 24 weeks, were used to assess HO. Fragments ≥ 3 mm in or around the capsule with a clear boundary from surrounding tissues, having a high density equivalent to that of bone, were defined to be valid free bone fragments (Fig. 2 A, B). In addition, only new bone fragments that appeared postoperatively were considered valid to exclude bone fragments that were present before surgery.

The Dorr classification [15] was used to evaluate the medullary cavity morphology of the femur. The femurs were classified as A, B, or C based on their shape and bone structure. Classically, type A femurs have the narrowest isthmus (the narrowest part of the femoral canal), whereas type C femurs have the widest, with the isthmus being defined as the narrowest part of the canal. Radiographic assessments were reviewed independently by two orthopedic trauma surgeons who assessed 92 randomized radiographs. The Kappa coefficient (for categorical data) for interobserver reliability was 0.74.

### Surgical procedure

The surgery was performed by a team of seven orthopedic surgeons with varying levels of experience ranging from 1 to 10 years. A conventional posterior approach was used in all cases to ensure a consistent surgical technique across the cohort. Hip stem selection was based on a comprehensive assessment of each surgeon’s preference and the complex morphology of the femoral marrow. Exeter hip stems (Stryker Orthopaedics, Mahwah, NJ, USA) were selected in 20 cases in which a cemented approach was deemed appropriate. In 72 cases, tapered wedge stems were used as cementless stems. Of these, INITIA stems (Kyocera, Kyoto, Japan) were used in 54 cases, Taperloc Complete Stems (Zimmer Biomet, Warsaw, IN, USA) in 11, and Accolade 2 Hip Stems (Stryker Orthopaedics, Nahawa, NJ, USA) in seven.

Significant attention was devoted to postoperative care, with all patients granted full weight bearing. This postoperative directive reinforces the successful outcome achieved by the meticulous execution of the surgical procedure.

### Clinical evaluation

The PMS and 11-point pain intensity numerical rating scale (NRS 11) were used to assess treatment outcomes at the final follow-up examination. The NRS 11 is based on an 11-point scale scored from 0, representing “no pain” to 10, representing “pain as bad as you can imagine” and “worst pain imaginable” [16].

### Statistical analysis

The patients were divided into two groups based on the presence (group P) and absence of HO (group A). Statistical analyses were performed using EZR software (Saitama Medical Center, Jichi Medical University, Saitama, Japan). An independent-sample *t*-test was used for continuous variables, whereas a chi-square test was used to compare categorical variables. In addition, a multivariate logistic regression analysis was performed using ectopic ossification as an objective variable. Differences with p < 0.05 were considered to be statistically significant.

## Results

The study population consisted of 92 patients (26 males and 66 females), with a mean age of 80.0 years (range, 61–95 years). The baseline characteristics of the patients are summarized in Table 1. The mean follow-up period was 22.9 months (range, 6–41 months).

**Table 1:** Patient demographics

	Group P	Group A	*p* value
Patients, *n*	50	42	
Mean age, yrs (SD)	80.4 (7.3)	79.6 (9.5)	0.68
Sex, *n* (%)			0.49
Men	16 (32)	10 (23.8)	
Women	34 (68)	32 (76.2)	
Mean BMI, kg/m^2^ (SD)	20.8 (4.1)	20.8 (3.6)	0.95
Pre-injury mobility, *n* (%)			0.22
Independent	33 (66)	21 (50)	
One aid	12 (24)	12 (28.6)	
Walker or frame	5 (10)	9 (21.4)	
Wheelchair	0	0	
Mean days to surgery (SD)	8.7 (5.8)	6.8 (5.4)	0.12
Dorr classification, *n* (%)			0.57
A	18 (36)	11 (26.1)	
B	30 (60)	30 (71.4)	
C	2 (4)	1 (2.4)	
ASA classification, *n* (%)			0.52
1	0	0	
2	31 (62)	29 (69)	
3	19 (38)	13 (31)	
4	0	0	
NSAID usage, *n* (%)	7 (14)	9 (21.4)	0.41

BMI, body-mass index; ASA, American Society of Anesthesiologists; HO, heterotopic ossification; NSAID, nonsteroidal anti-inflammatory drug

In this cohort, 50 (54%) patients developed HO. Among those affected, 24 were classified as class 1, 22 as class 2, and four as class 3, whereas no cases were classified as class 4. None of the patients exhibited clinically significant HO, and no surgical intervention involving HO resection was required. No significant differences in clinical outcomes were observed, including PMS and NRS scores, between the two groups (Table 2).

**Table 2:** Clinical outcomes and surgery-related factors

	Group P (*n* = 50)	Group A (*n* = 42)	*p* value
Mean pre-injury PMS (range; SD)	7.26 (4 to 9; 1.32)	7.00 (4 to 9; 1.62)	0.40
Mean PMS (range; SD)	6.24 (2 to 9; 2.04)	6.19 (2 to 9; 2.13)	0.91
Mean NRS (range; SD)	0.92 (0 to 7; 1.53)	0.74 (0 to 5; 1.17)	0.53
Mean operative time, min (SD)	79.4 (31)	66.3 (11.7)	0.01*
Mean blood loss, g (SD)	226 (136)	156 (87.8)	0.006*
Patients who had free bone fragments, *n* (%)	29 (58)	3 (7.1)	< 0.001*
Femoral component, *n* (%)			0.13
cementless stem	36 (72)	36 (85.7)	
cemented stem	14 (28)	6 (14.3)	

HO, heterotopic ossification; PMS, Parker Mobility Score; SD, standard deviation; NRS, numerical rating scale

*p < 0.05

The surgery-related factors are summarized in Table 2. There was no significant difference in the number of cemented cases between the two groups (*p* = 0.13). The operative duration and blood loss were greater in group P than in group A. The number of cases involving free bone fragments was significantly higher in group P than in group A (29/50[58%] versus [vs.] 3/42[7.1%]).

Logistic regression analysis revealed that the presence of free bone fragments was an independent factor related to the development of HO in patients who underwent bipolar HA (BHA) (odds ratio 23.4 [95% confidence interval 5.18–105]; *p* < 0.001) (Table 3).

**Table 3:** Multivariate analysis

	Odds ratio (95% CI)^+^	*p* value
Age	1.04 (0.97–1.12)	0.92
Gender	0.80 (0.22–2.92)	0.73
BMI	0.99 (0.85–1.16)	0.91
Mean days to surgery	0.97 (0.87–1.09)	0.62
Dorr classification	0.49 (0.14–1.67)	0.25
ASA classification	2.44 (0.69–8.69)	0.17
Mean operative time	1.03 (0.99–1.07)	0.11
Mean blood loss	1.00(1.00–1.01)	0.22
Patients who had free bone fragments	23.4 (5.18–105)	< 0.001*
Cemented stem	1.68 (0.34–8.43)	0.53

BMI, body-mass index; ASA, American Society of Anesthesiologists

*p < 0.05

^+^The Odds ratio is for present ossification as compared with absent ossification; the 95% confidence interval (CI) is given for the occurrence of HO

### Representative case

**Fig. 3:**
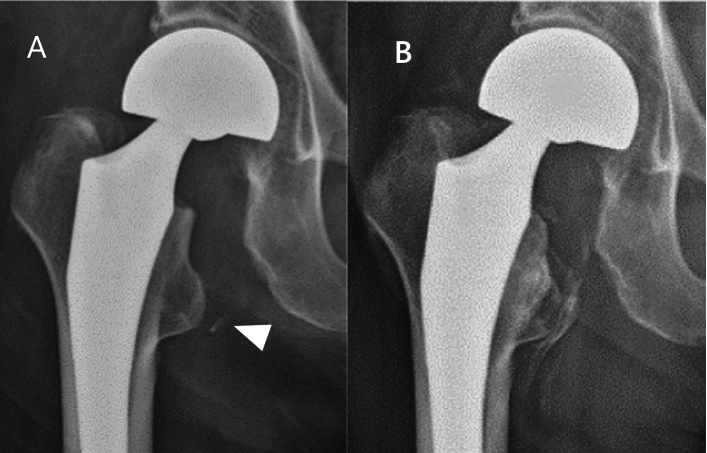
Representative case: 89 years-old male. **A** The immediate postoperative period. A 5 mm-sized free bone fragment is seen medially (arrowhead). **B** 24 weeks postoperatively. Class 3 heterotopic ossification is seen medially

An 89-year-old male sustained a fracture of the right femoral neck after falling while walking for which he underwent BHA on day 7. Free bone fragments were observed on the medial side of the hip joint (Fig. 3A, white arrowhead). At 24 weeks postoperatively, class 3 HO was evident on the medial side of the hip joint (Fig. 3B).

## Discussion

HO occurred in 54% of the patients in our cohort, with the majority of cases being Brooker class 1 or 2. The presence of free bone fragments was significantly associated with the development of HO in the patients who underwent HA. The incidence of HO in our cohort (54%) was higher than that reported in previous studies (0–28%). However, in these studies, HO was assessed at only one time point using anteroposterior radiography. In contrast, our study evaluated HO at four different time points (i.e., postoperatively and 2, 12, and 24 weeks after surgery) using both anteroposterior and lateral radiographs, which may have led to an overestimation. In addition, the number of patients using NSAIDs was relatively small. NSAIDs have the potential to prevent the development of HO [17], and a lack thereof could contribute to an elevated risk of HO.

The presence of free bone fragments was significantly associated with the development of HO in the patients who underwent HA. Histological studies have revealed that HO can be induced by both intramembranous processes, which do not involve cartilage formation and endochondral processes, which require a cartilage template [10]. The pathophysiology of HO involves excessive formation of ectopic cartilage, followed by endochondral bone formation, and is triggered by injury. During this process, free bone fragments may act as scaffolds for HO. HO pathogenesis is believed to result from the differentiation of mesenchymal cells into osteoprogenitor cells. To form heterotopic bone, there must be an inciting event, such as surgery, a supply of mesenchymal cells, such as bone debris, and a signal from the site of injury in an environment conducive to promoting heterotopic bone growth [18–20]. HO can be experimentally induced by trauma to soft tissues, transplantation of living cells, or bone extracts such as bone morphogenetic proteins [21]. Several investigators have attributed “bone dust” to the stimulation of HO [18, 22]. To prevent HO from free bone fragment(s), several previous studies have reported that massive intraoperative rinsing effectively prevents HO. Kantak et al. reported significantly less HO with rinsing using ≥ 3000 ml compared with rinsing using ≤ 1000 ml in 175 patients undergoing their first THA [5]. The importance of this surgical technique has been supported by Shields et al., who placed a plastic drape around the femoral head and neck, covering the soft tissues to collect and contain bone debris during femoral preparation, and reduced the formation of HO from 58.3 to 32.8%. Ahrengart et al. and Sell et al. suggested that clearing the surgical site of bone fragments contributes to lower HO rates. In our study, we considered new free bone fragments that appeared immediately after surgery to be significant; therefore, free bone fragments must have emerged during surgical manipulations such as femoral neck cutting and head removal.

Although no free bone fragments were observed under direct observation during the surgery, some may have been left behind. Therefore, careful palpation of the surgical field, use of drapes to prevent bone fragments, and adequate rinsing should be considered in future surgeries.

Some systematic reviews and meta-analyses have reported a higher risk of HO in HA with cement [9, 23]. Both the heat released by the polymerization of bone cement and its cytotoxicity may stimulate HO formation in the surrounding tissues. However, our study did not indicate that cement use was associated with the development of HO. The small sample size of this study may have contributed to the lack of significant differences.

Our study has several limitations, the first of which is its retrospective design and the possibility of selection bias, in addition to its low evidence level. The fact that the follow-up rate was only 50% may also have introduced a selection bias. Second, this was a short-term follow-up study. In vitro and animal studies have shown that the events involved in the development of ossification occur during the first few days following the procedure [19, 24]. Therefore, a long-term study may not be necessary to investigate the presence of HO. Third, although the data were collected from several cases, the sample size was relatively small. Fourth, we assessed functional outcomes based only on pre-injury mobility levels. This study involved a radiological review without clinical outcome assessment. Therefore, our conclusions are limited to radiographic analysis, and we are unable to comment definitively on the effect of our intervention on clinical outcomes. However, our study was not designed to compare the clinical outcomes. Fifth, we used only radiographs to evaluate free bone fragments, which may have resulted in an underestimation. Although additional evaluation using computed tomography would have been more precise, we believe that radiography, which can be performed early in the postoperative period, is a simple and realistic method. Sixth, the study population was exclusively Asian. The homogeneous background of patients undergoing HA may differ from that in other studies, resulting in different clinical outcomes. Seventh, clinical assessment was performed by orthopedic surgeons, which may have affected the results owing to observer bias. Finally, several femoral stems were used, and several operators performed the procedures, which may have confounded the results.

HO was observed in 54% of the study population. The multivariate analysis revealed that free bone fragments in the immediate postoperative period may trigger the development of HO. Nevertheless, further studies with larger sample sizes and long-term follow-up are warranted.

## References

[1] Hayashi D, Gould ES, Ho C, Caruana DL, Komatsu DE, Yang J et al (2019) Severity of heterotopic ossification in patients following surgery for hip fracture: A retrospective observational study. BMC Musculoskelet Disord. 10.1186/s12891-019-2725-7 31351447 10.1186/s12891-019-2725-7 PMC6661104

[2] Cobb TK, Berry DJ, Wallrichs SL, Ilstrup DM, Morrey BE (1999) Functional outcome of excision of heterotopic ossification after total hip arthroplasty. Clin Orthop Relat Res 361(131):139 10.1097/00003086-199904000-00018 10212606

[3] Neal B (2003) Effects of heterotopic bone formation on outcome after hip arthroplasty. ANZ J Surg 73:422–426. 10.1046/j.1445-2197.2003.02647.x 12801342 10.1046/j.1445-2197.2003.02647.x

[4] Ritter MA, Vaughan RB (1977) Ectopic ossification after total hip arthroplasty. Predisposing factors, frequency, and effect on results. The Journal of Bone Joint Surgery. 59(345):351 403193

[5] Kantak AP, Shah NN (2017) Extensive surgical wound lavage reduces the incidence and severity of heterotopic ossification in primary total hip replacement: A study of 175 hip replacements. Hip Pelvis 29:234. 10.5371/hp.2017.29.4.234 29250497 10.5371/hp.2017.29.4.234 PMC5729165

[6] Della Valle AG, Ruzo PS, Pavone V, Tolo E, Mintz DN, Salvati EA (2002) Heterotopic ossification after total hip arthroplasty: a critical analysis of the Brooker classification and proposal of a simplified rating system. J Arthroplasty 17(870):875. 10.1054/arth.2002.34819 10.1054/arth.2002.34819 12375245

[7] Publications Pvt Ltd M. IJOrtho_JAN_2008.indd. n.d.

[8] Corrigan CM, Greenberg SE, Sathiyakumar V, Mitchell PM, Francis A, Omar A et al (2015) Heterotopic ossification after hemiarthroplasty of the hip-a comparison of three common approaches. J Clin Orthop Trauma 6:1–5. 10.1016/j.jcot.2014.11.004 26549944 10.1016/j.jcot.2014.11.004 PMC4551151

[9] Elmenshawy AF, Salem KH (2021) Cemented versus cementless bipolar hemiarthroplasty for femoral neck fractures in the elderly. EFORT Open Rev 6:380–386. 10.1302/2058-5241.6.200057 34150332 10.1302/2058-5241.6.200057 PMC8183153

[10] Shimono K, Uchibe K, Kuboki T, Iwamoto M (2014) The pathophysiology of heterotopic ossification: current treatment considerations in dentistry. Japanese Dental Science Review 50:1–8. 10.1016/j.jdsr.2013.07.003

[11] Shields JS, Mofidi A, Ward WG, Jinnah RH (2011) Does a plastic drape reduce incidence of heterotopic ossification after hip resurfacing? Clin Orthop Relat Res 469:1606–1613. 10.1007/s11999-010-1606-z 20886321 10.1007/s11999-010-1606-z PMC3094620

[12] Parker MJ, Palmer CR (1993) A new mobility score for predicting mortality after hip fracture. The Journal of Bone and Joint Surgery British volume. 755(797):798 10.1302/0301-620X.75B5.8376443 8376443

[13] Brooker AF, Bowerman JW, Robinson RA, Riley LH (1973) Ectopic ossification following total hip replacement. Incidence and a method of classification. Journal of Bone and Joint Surgery Series A 55(1629):1632. 10.2106/00004623-197355080-00006 4217797

[14] Hug KT, Alton TB, Gee AO (2015) In brief: classifications in brief: Brooker classification of heterotopic ossification after total hip arthroplasty. Clin Orthop Relat Res 473:2154–2157. 10.1007/s11999-014-4076-x 25427427 10.1007/s11999-014-4076-x PMC4419015

[15] Dorr LD, Faugere M-C, Mackel AM, Gruen TA, Bognar B, Malluche HH (1993) Structural and cellular assessment of bone quality of proximal femur. Bone 14(231):242 10.1016/8756-3282(93)90146-2 8363862

[16] Hawker GA, Mian S, Kendzerska T, French M (2011) Measures of adult pain: visual analog scale for pain (VAS Pain), numeric rating scale for pain (NRS Pain), McGill pain questionnaire (MPQ), short-form mcgill pain questionnaire (SF-MPQ), Chronic pain grade scale (CPGS), short form-36 bodily pain scale (SF-36 BPS), and measure of intermittent and constant osteoarthritis pain (ICOAP). Arthritis Care Res (Hoboken). 10.1002/acr.20543 22588748 10.1002/acr.20543

[17] Kienapfe H (1999) Prevention of heterotopic bone formation after total hip arthroplasty: a prospective randomised study comparing postoperative radiationtherapy with indomethacin medication. Arch Orthop Trauma Surg 119:296–302 10447627 10.1007/s004020050414

[18] Ahrengart L. (1991) Periarticular heterotopic ossification after total hip arthroplasty: risk factors and consequences. Clin Orthop Relat Res. 1899637

[19] Chalmers J, Gray DH, Rush J (1975) Observations on the induction of bone in soft tissues. Journal of Bone and Joint Surgery Series B 57(36):45. 10.1302/0301-620x.57b1.36 1090627

[20] McCarthy EF, Sundaram M (2005) Heterotopic ossification: a review. Skeletal Radiol 34:609–619. 10.1007/s00256-005-0958-z 16132978 10.1007/s00256-005-0958-z

[21] Aho HJ, Aro H, Juntunen S, Strengell L, Michelsson JE (1988) Bone formation in experimental myositis ossificans. Light and electron microscopic study APMIS 96:933–940. 10.1111/j.1699-0463.1988.tb00965.x 2973337 10.1111/j.1699-0463.1988.tb00965.x

[22] Healy WL, Lo TC, DiSimone AA, Pfeifer BA (1995) Single-dose irradiation for the prevention of heterotopic ossification after total hip arthroplasty. A comparison of doses of five hundred and fifty and seven hundred centigray. J Bone Joint Surg Am 77:590–595 7713977 10.2106/00004623-199504000-00013

[23] Fu M, Shen J, Ren Z, Lv Y, Wang J, Jiang W (2023) A systematic review and meta-analysis of cemented and uncemented bipolar hemiarthroplasty for the treatment of femoral neck fractures in elderly patients over 60 years old. Front Med (Lausanne). 10.3389/fmed.2023.1085485 36817792 10.3389/fmed.2023.1085485 PMC9932906

[24] Vane JR (1971) Inhibition of prostaglandin synthesis as a mechanism of action for aspirin-like drugs. Nat New Biol 231(25):232 235. 10.1038/newbio231232a0 5284360 10.1038/newbio231232a0

